# Polyethylene nano- and microplastics trigger metabolic stress responses in human vaginal epithelial cells

**DOI:** 10.1038/s41420-026-03038-6

**Published:** 2026-03-24

**Authors:** Paola Pontecorvi, Matteo Cassandri, Alessandra Gianoncelli, Lorella Pascolo, Fabrizio Cece, Elena Niccolai, Simona Camero, Valentina Bonanni, Sara Bozzer, Enrico Romano, Simona Ceccarelli, Claudia Bearzi, Roberto Rizzi, Amedeo Amedei, Antonio Angeloni, Cinzia Marchese, Francesca Megiorni

**Affiliations:** 1 Department of Medical-Surgical Sciences and Biotechnologies, Sapienza University of Rome, Rome, Italy; 2 Department of Experimental Medicine, Sapienza University of Rome, Rome, Italy; 3 Elettra Sincrotrone Trieste, Basovizza, Trieste, Italy; 4 Institute for Maternal and Child Health, IRCCS Burlo Garofolo, Trieste, Italy; 5 Department of Experimental and Clinical Medicine, University of Florence, Florence, Italy; 6 Department of Life Sciences, Health and Health Professions, Link Campus University, Rome, Italy; 7 Department of Sense Organs, Sapienza University of Rome, Rome, Italy; 8 Institute for Biomedical Technologies, National Research Council, Segrate, Italy; 9 Department of Well-being, Health and Environmental Sustainability, Sapienza University of Rome, Rieti, Italy

**Keywords:** Cellular imaging, Transcription

## Abstract

Nano- and microplastics (N/MPs) are emerging environmental contaminants increasingly detected in multiple human tissues, yet their biological effects remain poorly defined. The vaginal epithelium represents a relevant but largely unexplored site of exposure. Here, we investigated the metabolic and elemental responses of human vaginal keratinocytes (VK2 E6/E7) exposed to polyethylene (PE) N/MPs using an integrated transcriptomic and synchrotron imaging approach. Cells were challenged with environmentally relevant unlabeled PE N/MPs (200 nm - 9 µm) and with traceable PE quantum dot-labeled nanoparticles (PE QDs/NPs). NanoString nCounter analysis revealed widespread transcriptional alterations across metabolic processes, with activation of pro-inflammatory and oxidative stress pathways, dysregulation of lipid metabolism, and impaired cholesterol biosynthesis. Immune-related transcripts suggested the emergence of a tolerogenic and immunomodulatory phenotype. Complementary scanning transmission X-ray microscopy and low-energy X-ray fluorescence mapping confirmed substantial nanoparticle internalization and revealed intracellular carbon accumulation, increased oxygen signals, and altered sodium and magnesium distributions, consistent with ionic and membrane perturbations. Collectively, these findings indicate that PE N/MPs might elicit profound metabolic stress in vaginal epithelial cells, including redox disequilibrium and immune modulation, possibly driving them toward an adaptive but inflammation-linked phenotype. While the broader implications for epithelial barrier function and mucosal homeostasis require further validation in more complex models, this study provides a mechanistic framework to explore how environmental polymeric contaminants may influence vaginal epithelial cell physiology.

## Introduction

Plastic pollution has become a pervasive environmental and public health concern, with microplastics (MPs < 5 mm) and nanoplastics (NPs < 1 µm) increasingly detected across ecosystems, including the human body [1]. These plastic particles, originating from degradation of larger plastic debris or primary sources, such as cosmetics and personal care products, have been found in various biological fluids and tissues, including blood, brain, placenta, and even ovarian follicular fluid [2–5]. Their ability to enter the human body and interact with cells has raised concerns about their potential impact on health, mainly at the epithelial interfaces that serve as primary barriers to external agents [6, 7]. Among plastic polymers, polyethylene (PE) is one of the most widely produced and persistent types, with increasing evidence suggesting its bioavailability and potential toxicity at the nano- and microscale [8, 9]. Due to their small size, N/MPs can breach epithelial barriers and accumulate in tissues, eliciting oxidative stress, inflammation, and disruption of lipid homeostasis [10–12]. However, while numerous studies have explored the effects of N/MPs in gastrointestinal and respiratory models [13], recent attention has turned to the female reproductive tract [14], which may be directly exposed to PE N/MPs through the use of menstrual products, lubricants, and other intravaginal devices [15]. Despite these plausible exposure routes, the N/MPs effects on vaginal epithelial cells, mostly keratinocytes, have been largely unexplored.

In a recent study, our group addressed this critical gap by demonstrating that PE N/MPs (200 nm to 9 µm) are efficiently internalized by human vaginal keratinocytes and can induce cytotoxicity, apoptosis, and structural alterations [16]. Notably, we also found that, even when cells recovered from acute exposure in terms of viability and morphology, epigenetic alterations—particularly in the expression of DNA methyltransferases and demethylases—persisted, potentially predisposing cells to inflammation, accelerated ageing, or malignant transformation [16].

However, the molecular mechanisms underlying these effects remain poorly defined. To tackle this issue, we employed the NanoString nCounter Metabolic Pathways Panel to comprehensively profile the transcriptional landscape of vaginal keratinocytes following PE N/MP exposure, focusing on genes involved in core metabolic pathways and immunometabolism, key, but underexplored, regulators of epithelial homeostasis [17]. This high-throughput, amplification-free technology, coupled with Gene Set Enrichment Analysis (GSEA), allowed us to capture how PE N/MP exposure reshapes metabolic and stress-response pathways in vaginal keratinocytes, including immune-like metabolic reprogramming relevant to inflammation and barrier dysfunction. In the context of epithelial interactions with environmental particulates, the term “metabolic stress/reprogramming” is used here to describe a coordinated but multifaceted cellular response encompassing redox imbalance and oxidative stress-related pathways, lipid metabolic dysregulation, alterations in energy-related processes, and proteostasis-associated stress signatures, rather than a single uniform process.

To complement molecular analyses, we employed synchrotron radiation-based soft X-ray imaging [18], to achieve high-resolution, label-free spatial mapping of cellular ultrastructure and elemental composition, allowing the tracking of elemental redistribution within complex metabolic pathways [19].

In this study, we first exposed VK2 E6/E7 vaginal cells to unlabeled PE N/MPs to model realistic environmental exposure. However, to gain deeper insight into cellular uptake dynamics and the intracellular fate of NPs, particularly given their heightened biological reactivity [20], we employed synthesized cadmium selenide (CdSe) quantum dot (QD)-labeled PE NPs (PE QDs/NPs) with controlled size distribution and stable QD core inclusion, enabling dual-mode detection via fluorescence and elemental imaging [21]. Following the strategy outlined by Zingaro et al. [12] and Gianoncelli et al. [14], we combined scanning transmission X-ray microscopy (STXM) with low-energy X-ray fluorescence (LEXRF) to map at sub-micron resolution the intracellular distribution of PE QDs/NPs, while simultaneously correlating their localization with elemental signatures and structural changes in the exposed cells.

Using sensitive molecular and imaging approaches, we demonstrate that environmental plastic particles may compromise vaginal epithelial function through metabolic reprogramming, thereby predicting relevant implications for both women’s health and environmental toxicology.

## Results

### Oxidative stress and immune response activation, lipid and energy metabolism dysregulation in VK2 E6/E7 cells exposed to PE N/MPs

**Fig. 1:**
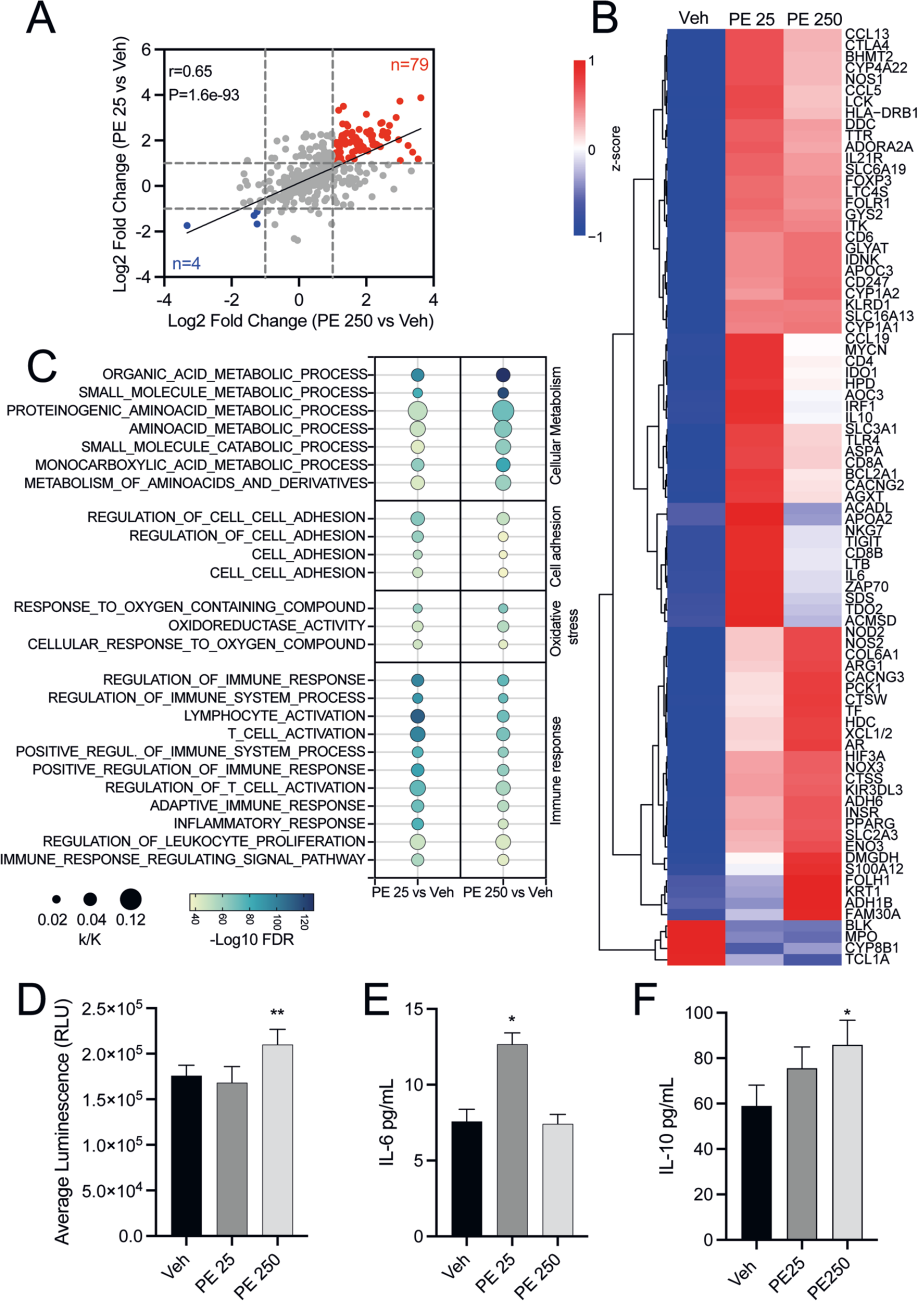
PE N/MP exposure promotes metabolic reprogramming in VK2 E6/E7 cells. **A** Correlation of differential gene expression between VK2 E6/E7 cells treated with 25 µg/mL (PE 25) or 250 µg/mL (PE 250) for 48 h. Data are reported as log_2_(Fold change). Red dots indicate commonly upregulated genes (*n* = 79), while blue dots indicate commonly downregulated genes (*n* = 4) across conditions. Pearson coefficient (r) and p-value (P) are reported in the figure. **B** Cluster heatmap of upregulated and downregulated genes identified in (**A**). Expression values are expressed as z-score. The color legend is provided in the figure. **C** Bubble plot depicting GSEA performed on commonly upregulated genes in VK2 E6/E7 treated with 25 µg/mL (PE 25) or 250 µg/mL (PE 250) for 48 h. Enrichment significance is reported as -log_10_(FDR) and indicated with color gradient. Gene ratio of upregulated genes to total genes in enriched pathways (k/K) is reported as bubble size. **D** Bioluminescence-based ROS detection assay performed on supernatants of VK2 E6/E7 treated with 25 µg/mL (PE 25) or 250 µg/mL (PE 250) for 48 h (*n* = 3 ** *p* < 0.005, two-tailed unpaired Student’s t-test. Veh vs PE 250: mean difference +34283 units; 95% CI + 15862 to +52705; *p* = 0.002). **E** Quantification of IL-6 levels in supernatants of VK2 E6/E7 exposed to 25 µg/mL (PE 25) or 250 µg/mL (PE 250) for 48 h (*n* = 3 * *p* < 0.05, two-tailed unpaired Student’s t-test. Veh vs PE 25: mean difference +5.086 units; 95% CI + 1731 to +8441; *p* = 0.0227). **F** Quantification of IL-10 levels in supernatants of VK2 E6/E7 exposed to 25 µg/mL (PE 25) or 250 µg/mL (PE 250) for 48 h (*n* = 3 * *p* < 0.05, two-tailed unpaired Student’s t-test. Veh vs PE 250: mean difference +26.91 units; 95% CI + 4.118 to +49.69; *p* = 0.0306). Veh: cells exposed to Tween20 0.0025% solution. Asterisks indicate statistically significant differences between treated cells and the corresponding control.

Although data are still limited, increasing evidence shows that N/MPs can be internalized by human cells and, upon acute or chronic exposure, induce epigenetic and gene expression changes, which may underlie the disruption of multiple cellular processes [16, 22]. Thus, to explore how N/MP internalization impacts gene transcription in human vaginal keratinocytes, we employed NanoString technology through the nCounter Metabolic Pathways Panel. This approach enables the parallel quantification of 768 metabolism-associated genes, encompassing pathways related to energy generation, biosynthetic processes, molecular breakdown, cellular stress responses, and signal transmission. We exposed human immortalized vaginal keratinocytes, VK2 E6/E7 to 25 µg/mL (PE 25) and 250 µg/mL (PE 250) of a mixture of PE nano- and microspheres (200 nm–9 µm) for 48 hours (h), and then we defined the transcriptomic profile. Interestingly, we observed a strong and statistically significant positive correlation between the two treatment concentrations, indicating the activation of a transcriptional program that appears to be related to a specific N/MP concentration (Fig. 1A). Indeed, we identified 79 genes that were consistently upregulated and only 4 genes that were downregulated across conditions. These findings suggest that exposure to PE N/MPs predominantly induces transcriptional activation, while having a limited effect on transcriptional repression. Specifically, acute exposure (48 h) of VK2 E6/E7 to PE N/MPs at low concentrations (PE 25) activated inflammatory and redox signaling pathways (IL-6, IL-10, CCL13, TLR4, CYP1A1, NOX1/3/4), accompanied by compensatory responses involving lipid metabolism (PPARG, ACADL, APOC3) and antioxidant defenses (PPARGC1A, PRKAA2). Higher doses (PE 250) intensified these responses, with markers of chronic inflammation (TNF, IL-6, CCL4/5/19/13), enhanced NADPH oxidase activity (CYBB/NOX2, NOX3), upregulation of detoxification-related enzymes (FDXR, CYPs), and extensive reprogramming of lipid and energy metabolism (PPARG, SREBF2, SLC27A1, ACSF3, PCK1, ACADL, ACOT12) (Additional file 1: Fig. S1).

In addition, we performed a cluster analysis on the 83 genes commonly deregulated under both PE 25 and PE 250 exposures, which revealed the emergence of four distinct clusters based on the observed gene expression changes (Fig. 1B). Specifically, we identified one cluster of genes that encompass similar upregulation by both PE 25 and PE 250 doses, a second cluster that shows stronger upregulation in VK2 E6/E7 cells treated with the N/MP lower concentration, and a third cluster in which genes are more highly upregulated with the PE 250 dose compared to PE 25. Additionally, we identified a small cluster of four genes (BLK, MPO, CYP8B1 and TCL1A) that were consistently downregulated under both exposure conditions, showing an overlapping trend compared to vehicle (Veh)-treated cells. These findings further suggest that different doses of N/MPs modulate the same set of genes, but to varying extents.

Furthermore, we performed a GSEA on the 79 commonly upregulated genes, which revealed a significant enrichment in pathways indicative of metabolic stress (Fig. 1C) in cells exposed to PE N/MPs with respect to Veh. In detail, in silico analyses showed an increased expression of genes involved in processes related to organic acids and monocarboxylic acids, suggesting a reorganization of lipid metabolism [23]. In addition, we observed changes in gene expression related to amino acid processing, further supporting the hypothesis that N/MP exposure can disrupt metabolic homeostasis and contribute to bioenergetic imbalance in vaginal keratinocytes. Moreover, among the enriched pathways, we identified the “response to reactive oxygen species (ROS)”, consistent with evidence that N/MP exposure enhances ROS production and associated oxidative damage, including DNA lesions [24]. GSEA analyses highlighted enrichment in “cell adhesion”, which may indicate the induction of changes related to cell-cell and cell-matrix interactions that could impact epithelial integrity, with potential implications for the barrier function of keratinocytes [25]. Finally, among the most enriched classes, we identified pathways involved in the activation of immune processes, which may suggest that N/MPs are perceived as harmful stimuli capable of triggering defense mechanisms, even in a cellular context that is not primarily immune. These inflammatory signals may be part of a sterile para-inflammatory response, typically associated with exposure to exogenous materials [26]. The genes contributing to these enriched pathways and biological processes, as identified by GSEA, are depicted in detail in Additional file 1: Fig. S2.

To functionally validate the transcriptional changes in ROS pathways, intracellular hydrogen peroxide (H₂O₂) levels were quantified using the ROS-Glo™ H₂O₂ assay. No significant increase in H₂O₂ levels was detected in VK2 E6/E7 cells exposed to PE 25 compared to Veh-treated controls, whereas a significant accumulation of H₂O₂ was observed following exposure to PE 250 (Fig. 1D). Together, these data might indicate that low-dose PE N/MP exposure induces redox-related transcriptional remodeling without measurable oxidative stress, while high-dose exposure exceeds cellular antioxidant buffering capacity, resulting in detectable H₂O₂ accumulation.

Given the transcriptional evidence of immune pathway activation, we next evaluated whether PE N/MP exposure affected the secretion of the pro-inflammatory cytokine interleukin-6 (IL-6) and the anti-inflammatory cytokine interleukin-10 (IL-10). Enzyme-linked immunosorbent assay (ELISA) was performed to quantify IL-6 and IL-10 levels in culture supernatants after 48 h of PE N/MP exposure. As shown in Fig. 1E, analysis of IL-6 secretion confirmed the transcriptional trends observed with NanoString. Exposure to a low concentration of PE N/MPs (PE 25) resulted in a significant increase in IL-6 protein levels compared to Veh-treated controls, consistent with the activation of inflammatory pathways at the transcriptional level. In contrast, exposure to a higher concentration (PE 250) yielded IL-6 levels comparable to Veh, suggesting that at elevated doses, the engagement of compensatory immunomodulatory pathways may regulate and constrain the inflammatory response. Indeed, Fig. 1F shows that PE N/MPs induced a progressive increase in IL-10 secretion compared to Veh-treated cells. While the increase observed at the lower concentration (PE 25) did not reach statistical significance, cells exposed to the higher dose (PE 250) displayed a significant elevation in IL-10 protein levels compared to Veh-treated controls. This discrepancy with NanoString data may reflect post-transcriptional regulation and the inherent differences between mRNA expression and cumulative protein secretion measurements.

### PE QDs/NPs accumulated within perinuclear vesicles in human VK2 E6/E7 cells after acute exposure

**Fig. 2:**
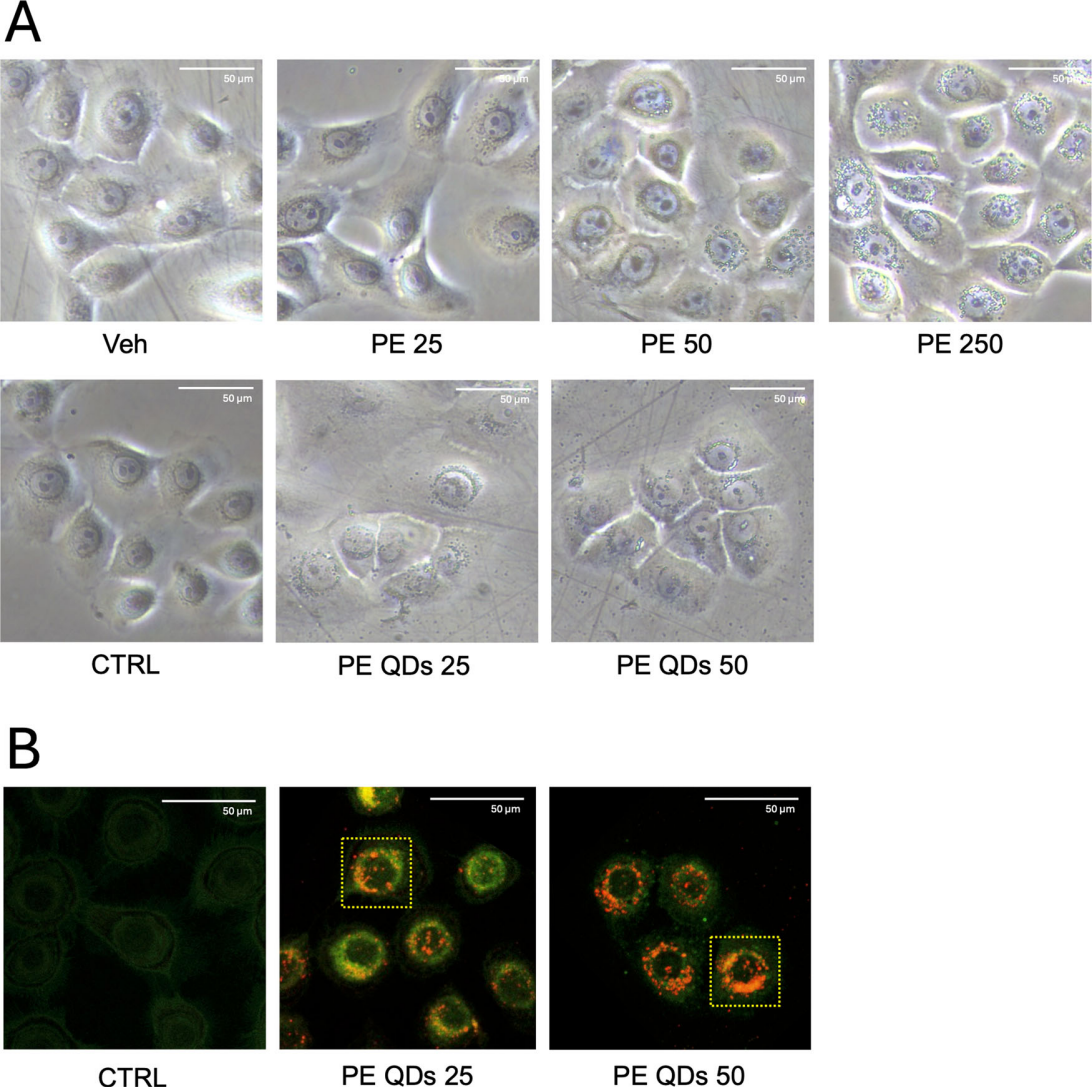
PE N/MPs and PE QDs/NPs perinuclearly accumulate in VK2 E6/E7 cells after acute exposure. **A** VK2 E6/E7 cells exposed to unlabeled PE N/MPs (25–250 μg/mL; 200 nm–9 μm) for 48 h showed dose-dependent vesicle formation. Vesicles were sparse and diffuse at 25 and 50 μg/mL (PE 25, PE 50), while 250 μg/mL (PE 250) induced marked perinuclear accumulation. Exposure to labeled PE QDs/NPs (25–50 μg/mL; 200–300 nm) led to prominent perinuclear vesicles even at 25 μg/mL (PE QDs 25). Veh: cells exposed to Tween20 0.0025% solution. CTRL: unexposed cells. 40x magnification, scale bar = 50 μm. **B** Confocal microscopy confirmed QD fluorescence within vesicles, indicating PE NP internalization (red dots). Green cell autofluorescence aided cell morphology visualization. Yellow-boxed cells indicate those selected for synchrotron-based imaging (shown in Fig. 3B, C). 60x magnification, scale bar = 50 μm.

Considering the potentially greater reactivity of nanoparticles over microparticles (attributable to their smaller size and higher surface area-to-volume ratio, enhanced ability to penetrate the epithelial barrier, increased cellular internalization, and greater intracellular bioavailability) we decided to compare the morphological effects of unlabeled PE N/MPs (a mixture of 200 nm–9 μm particles) and labeled PE QDs/NPs (200–300 nm) on VK2 E6/E7 cells. Although fluorescently labeled NPs are commonly employed for biodistribution studies, their application is limited by photobleaching, aggregation, and low stability in biological environments [12], so we used PE QDs/NPs that show enhanced dispersion in biological media and stably emit in the red spectrum, rendering them well-suited for advanced imaging applications. For PE N/MPs, we selected three concentrations (25, 50, and 250 μg/mL), based on the findings of Gopinath et al. [27], Schmidt et al. [28] on human and murine skin keratinocytes, and our previous report [16]. For PE QDs/NPs, we adopted the experimental setup described by Zingaro et al. [12], testing two concentrations: 25 and 50 μg/mL. Although 250 μg/mL was initially considered, it was finally excluded from the main analyses for PE QDs/NPs, as it represents an excessively high dose that we used to accentuate biological effects and facilitate the detection of the mixture of PE N/MP. After 48 h of exposure to unlabeled PE N/MPs, VK2 E6/E7 exhibited morphological alterations, most notably the appearance of vesicular structures, with respect to Veh-treated cells. At low concentrations (PE 25 and PE 50), these vesicles were infrequent and diffusely distributed throughout the cytoplasm. In contrast, their number significantly increased at PE 250, displaying a distinct perinuclear accumulation that confirms findings from our previous work [16] (Fig. 2A). Notably, exposure to PE QDs/NPs led to perinuclear vesicles accumulation even at the lowest concentration tested (PE 25), clearly visible under standard optical microscopy, suggesting that nanoscale particles are preferentially internalized by cells. This observation is consistent with our prior transmission electron microscopy (TEM) analyses, which identified this size range as the most readily taken up [16].

When analyzed by confocal microscopy, the perinuclear vesicles of VK2 E6/E7 exposed to PE QDs/NPs emitted strong red fluorescence from the QDs, confirming that the particles were not simply attached to the cell surface but were indeed internalized and localized within the cytoplasm. The intrinsic green autofluorescence of VK2 E6/E7 cells facilitated their localization and morphological identification (Fig. 2B). Light fluorescence microscopy was preliminarily performed to detect the intracellular localization of QDs/NPs and to guide the selection of three to four representative cells per sample for high-resolution investigation using synchrotron radiation.

### Morphological and elemental distribution changes in VK2 E6/E7 cells following PE N/MP and PE QD/NP acute exposure

**Fig. 3:**
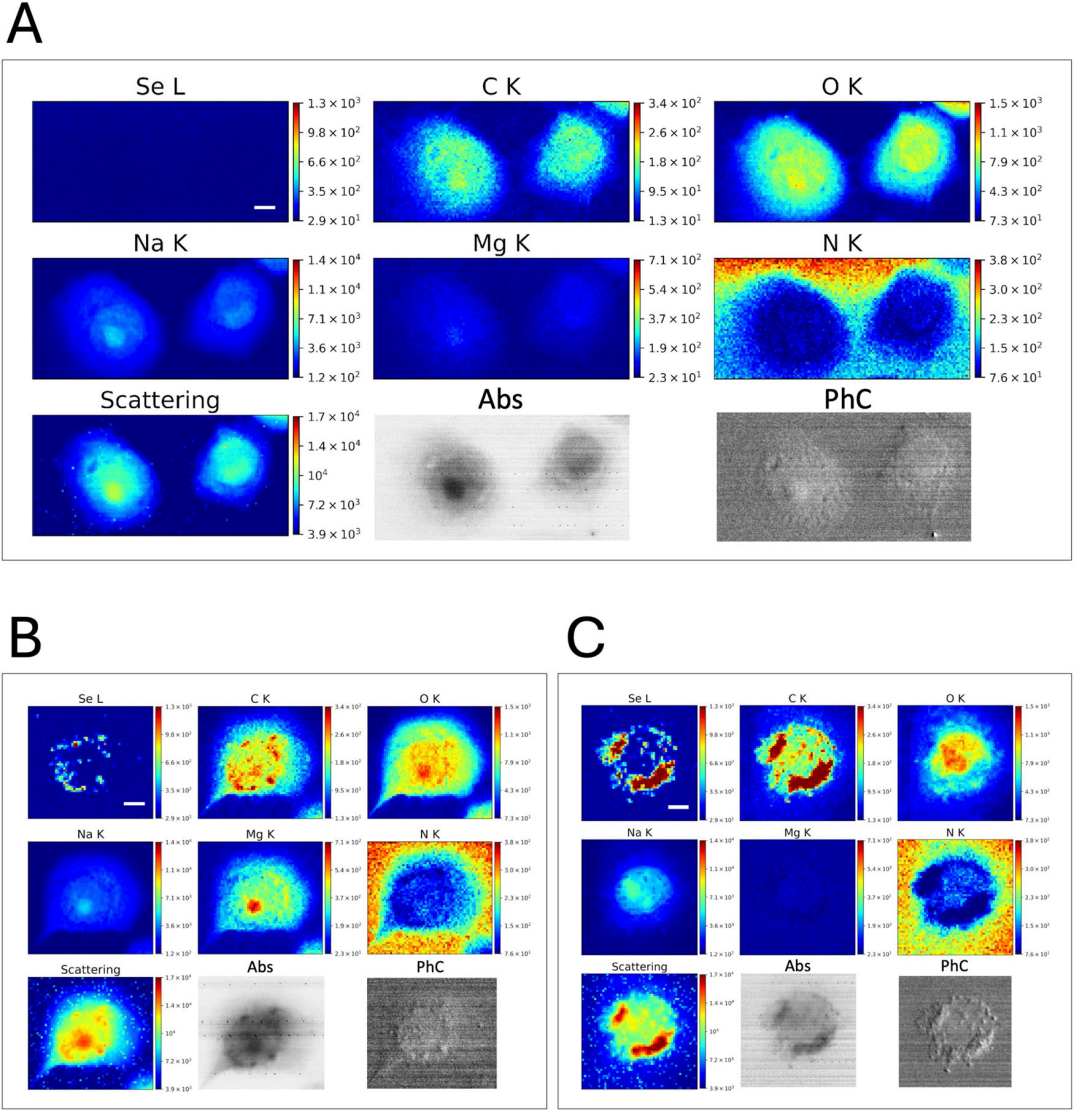
Morphological and elemental distribution alterations in VK2 E6/E7 cells after acute exposure to PE QD/NPs. Representative XRF distribution maps of Se, C, O, Na, Mg, and N, shown together with the corresponding photon scattering map and absorption (Abs) and differential phase contrast (PhC) STXM images of control cells (**A**) and cells exposed to 25 μg/mL (**B**) and 50 μg/mL (**C**) PE QDs/NPs. While XRF maps were collected at 1.7 keV with a spot size of 500 nm, STXM images were acquired at 1.5 keV at higher resolution (250 nm). The cells depicted in (**B**) and (**C**) correspond to the ones highlighted with yellow boxes in Fig. 2B, central and right panels, respectively. Scale bars = 5 μm (on Se channel).

**Fig. 4:**
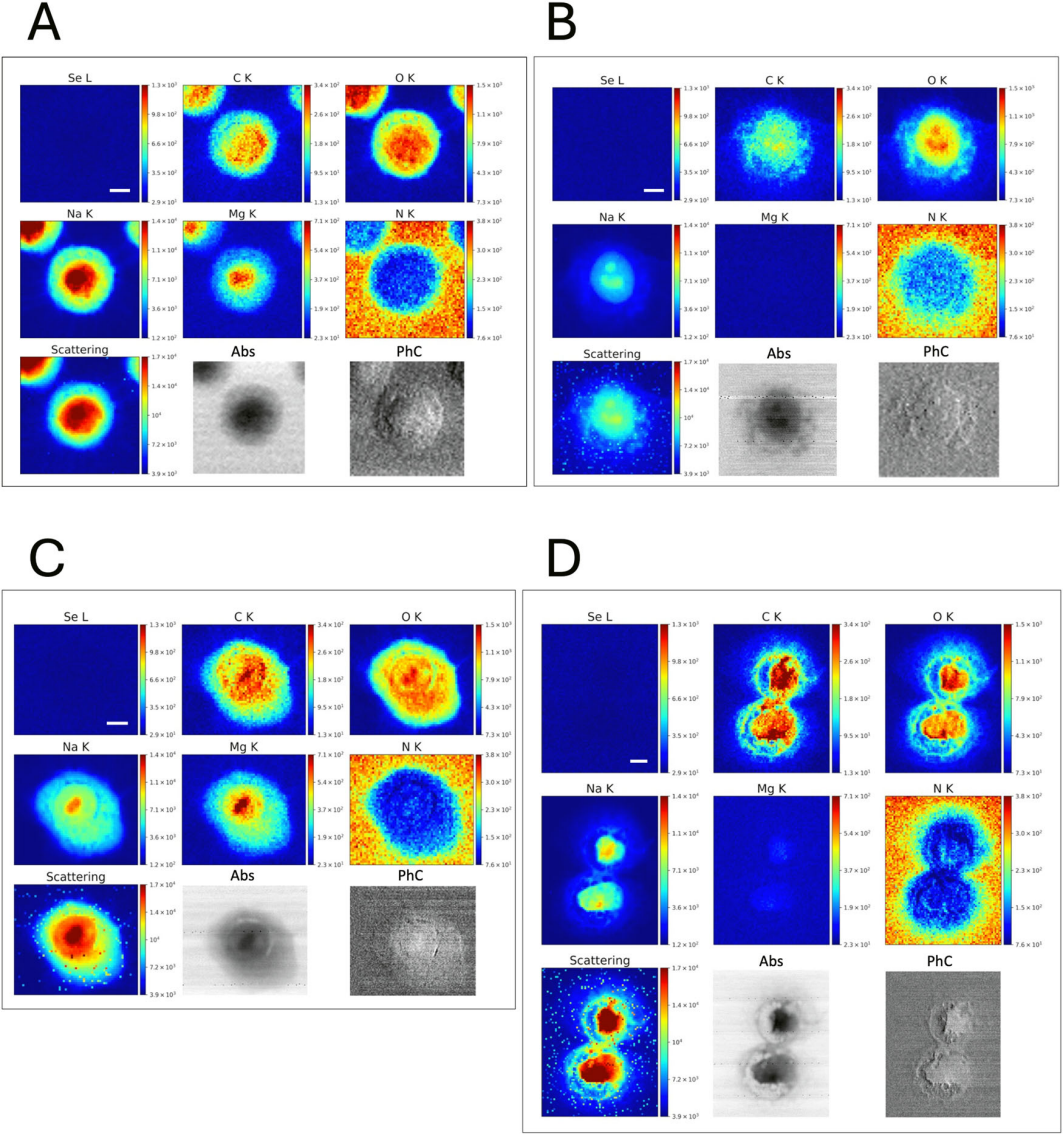
Acute PE N/MP exposure induced morphological changes and altered elemental profiles in VK2 E6/E7 cells. Representative XRF distribution maps of Se, C, O, Na, Mg, and N, shown together with the corresponding photon scattering map and absorption (Abs) and differential phase contrast (PhC) STXM images of control cells, namely Veh (**A**), and cells exposed to 25 μg/mL (**B**), 50 μg/mL (**C**) and 250 μg/mL (**D**) PE N/MPs. While XRF maps were collected at 1.7 keV with a spot size of 500 nm, STXM images were acquired at 1.5 keV at higher resolution (250 nm). Scale bars = 5 μm (on Se channel).

To investigate the NPs effects on cellular morphology and metabolism, and to simultaneously track their intracellular localization in vaginal keratinocytes, synchrotron radiation-based analyses were conducted. LEXRF was employed to confirm the presence of PE QDs/NPs by detecting the Se L-line emission arising from selenium (Se; a key component of the QDs), thus enabling the visualization of particle distribution at subcellular resolution. STXM absorption (Abs) and phase contrast (PhC) imaging provided morphological information on VK2 E6/E7 cells following 48 h exposure to PE QDs/NPs (Fig. 3B, C) and PE N/MPs (Fig. 4B–D), as well as their respective controls (Figs. 3A and 4A). The Abs contrast is determined by variations in density and/or thicknesses, thus it mainly highlights the cell nucleus, while the differential PhC is more sensitive to borders and structures, and thus, it better highlights the cell topography. Indeed, the PhC better identifies the cell cytoplasm which on the other hand appears relatively transparent in the Abs image. Unexposed cells displayed typical morphology with a more absorbent nuclear region, whereas cells exposed to PE QDs/NPs consistently showed bright vesicular structures in both Abs and PhC images, similar to those observed with confocal imaging as illustrated in Fig. 2B. However, unlike fluorescence microscopy, STXM alone, being a bidimensional technique, does not allow the visualization of intravesicular NP aggregates. LEXRF performed at 1.7 keV enabled the reliable detection of Se, confirming the intracellular presence of QDs/NPs. The Se distribution in Fig. 3B, C aligned well with the red fluorescence signals depicted in Fig. 2B, further supporting NP localization. Although STXM and LEXRF do not provide three-dimensional information, the discrete, nanometric Se hotspots are consistent with cytoplasmic accumulation. Control analyses, performed under identical conditions on unexposed cells, confirmed the specificity of the Se signal, as no Se-related fluorescence was detected (Figs. 3A and 4A).

Elemental maps of low-Z elements, such as carbon (C), oxygen (O), sodium (Na), magnesium (Mg), and nitrogen (N), were also simultaneously acquired to assess overall cellular integrity and co-localization patterns. Interestingly, PE QD/NP-treated cells (Fig. 3B, C) exhibited elevated C levels (co-localized with Se signal) compared to controls, likely mirroring the intracellular accumulation of PE-based polymers or possibly suggesting accumulation of lipid and other endogenous macromolecules. Potentially, proteotoxic stress likely caused by NPs can also impair protein degradation, leading to the build-up of misfolded or aggregated proteins enriched in C [29]. Additional analyses would be necessary to unambiguously determine the nature of C accumulation. We also observed a change in O content and distribution, which is in line with the morphological modification observed by light microscopy and in the STXM images. Moreover, in cells exposed to PE QDs/NPs, Na and Mg signals suggest possible alterations in the homeostasis of these elements, with interference with ion transport mechanisms and membrane potential regulation. The N signal is mainly coming from the sample support, *i.e*. silicon nitride (Si₃N₄) membranes; it is thus complementary to C and O arising from the cell itself and acts as an internal control for cell localization and detection.

In the case of PE N/MPs, particle distribution could not be tracked due to the absence of labeling and to their composition similar to the cell matter itself; therefore, these techniques did not allow confirmation of the internalization of the nanometric fraction of the PE mixture. Nevertheless, compared to control cells (Fig. 3A) and Veh-exposed ones (Fig. 4A), we were able to observe morphological alterations and intracellular C redistribution in VK2 E6/E7 cells exposed to increasing concentrations of PE N/MPs (Fig. 4). This was mainly evident for PE 250 (Fig. 4D), where cell morphology is highly altered compared to Veh, suggesting a stress-induced modification that might reflect LD accumulation, as previously suggested by NanoString results and by previous findings [12]. Altogether, these findings further underscore the alteration of cellular metabolism underlined by the extensive transcriptional analyses. Notably, Veh-treated cells exhibited an elemental distribution pattern similar to that observed in PE 50-exposed cells, suggesting that Tween20 alone, although used at non-toxic concentrations as confirmed in parallel experiments, could contribute to subtle variations in elemental mapping. While the biological relevance of this observation remains unclear, it highlights the importance of carefully considering vehicle-related effects in elemental imaging analyses.

### PE N/MPs and PE QDs/NPs induce lipid droplet deposition in VK2 E6/E7 cells

**Fig. 5:**
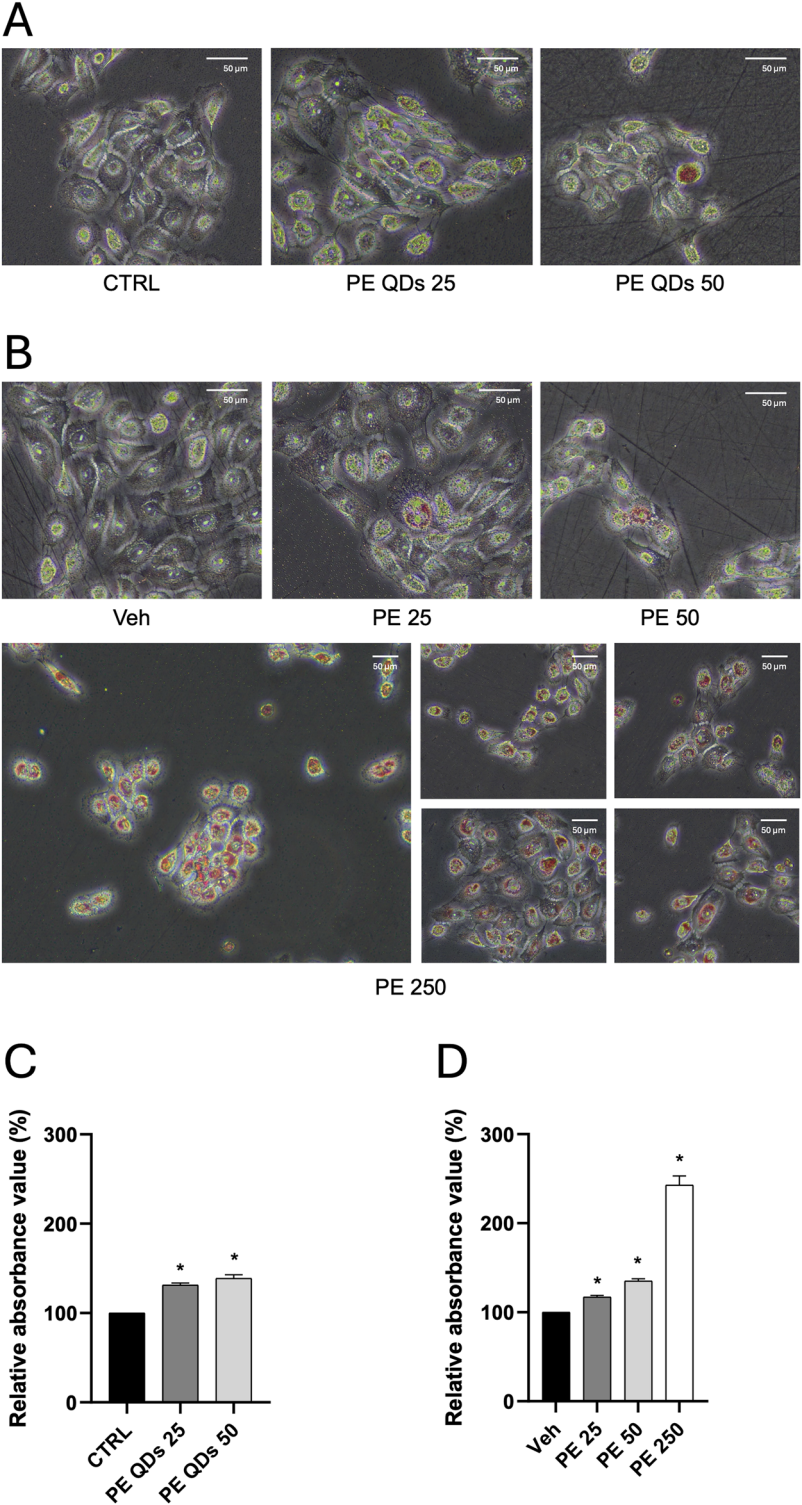
PE QDs/NPs and PE N/MPs induce concentration-dependent LDs accumulation in VK2 E6/E7 cells. **A**, **B** Representative images of Oil Red O staining of VK2 E6/E7 cells exposed for 48 h to PE QDs/NPs (25 and 50 μg/mL) and PE N/MPs (25, 50, and 250 μg/mL), respectively. Red dye deposition corresponds to intracellular lipid accumulation. Images were acquired at 40× magnification and 20x (for PE 250). Scale bar = 50 μm. **C**, **D** Quantification of LD content relative to controls. A significant, concentration-dependent increase in lipid droplet accumulation was observed following PE QD/NP and PE N/MP exposure, with the most pronounced effect at 250 μg/mL (PE 250), where nearly all cells showed abundant lipid deposition. Veh: cells exposed to Tween20 0.0025% solution; CTRL: unexposed cells. Asterisks indicate statistically significant differences between treated cells and the corresponding control (*n* = 3; *p < 0.05 two-tailed unpaired Student’s t-test. CTRL vs PE QDs 25: mean difference +31.27 units; 95% CI + 10.73 to +51.81; *p* = 0.0329. CTRL vs PE QDs 50: mean difference +38.75 units; 95% CI + 1.751 to +75.76; *p* = 0.0477. Veh vs PE 25: mean difference +17.14 units; 95% CI + 1.475 to +32.80; *p* = 0.0457. Veh vs PE 50: mean difference +35.17 units; 95% CI + 12.24 to +58.11; *p* = 0.0326. Veh vs PE 250: mean difference +142.9 units; 95% CI + 50.50 to +235.2; *p* = 0.0324).

Building on transcriptomic evidence indicating alterations in lipid metabolism and differential expression of genes involved in lipid droplet (LD) biogenesis, and on synchrotron-based imaging, Oil Red O staining was carried out to functionally validate and visualize LD accumulation in VK2 E6/E7 cells exposed to increasing concentrations of PE N/MPs and PE QDs/NPs. Cells were treated with PE QDs/NPs (25 and 50 μg/mL) and PE N/MPs (25, 50, and 250 μg/mL) (Fig. 5A and 5B, respectively), and LDs were quantified relative to control conditions (unexposed and Veh-treated cells; Fig. 5C, D). After 48 h of exposure, PE treatment led to a statistically significant, concentration-dependent increase in LD content (*p* < 0.05). Particularly striking was the response to PE 250 (Fig. 5D), where nearly all cells exhibited marked LD deposition. Our findings suggest that PE NPs, especially those stabilized with anionic surfactants, can strongly disrupt lipid homeostasis in vaginal keratinocytes, consistent with observations reported in other cellular models [12, 30].

## Discussion

N/MPs are widespread environmental contaminants, to which humans are continuously exposed through ingestion, inhalation, and dermal contact with consumer products [15, 27]. Accumulating evidence shows that they can deposit in tissues and trigger toxic responses across multiple organ systems, raising concerns about their long-term health effects [31]. In this context, we sought to characterize the impact of N/MP exposure on keratinocytes constituting the mucosal surfaces of the vaginal epithelium, which represents a low-explored yet clinically relevant target. In this study, we focused on acute exposure to PE N/MPs (48 h), as it represents the critical window in which the most substantial molecular and cellular alterations emerge, while our previous work also showed that under chronic exposure (21 days) VK2 E6/E7 cells can gradually recover morphology and viability, highlighting their adaptive capacity [16]. Herein, our NanoString profiling of VK2 E6/E7 cells exposed to PE N/MPs revealed a dose-dependent transcriptional activation, characterized by the upregulation of genes involved in immunometabolic regulation, redox signaling, as well as lipid and amino acid metabolism. Notably, low-dose exposure (PE 25) predominantly elicited a compensatory metabolic response, whereas higher concentrations (PE 250) led to a pronounced shift toward a stress-driven, dysfunctional, and potentially pathogenic transcriptional profile - consistent with the N/MP toxicity we previously proposed [16].

The most relevant findings involve lipid metabolism, where the simultaneous downregulation of SREBF2 and upregulation of PPARG in VK2 E6/E7 cells exposed to PE 250—though seemingly counterintuitive—may suggest a stress-induced metabolic shift aimed at restoring cellular homeostasis. SREBF2, a central regulator of cholesterol biosynthesis, controls genes involved in sterol and membrane lipid production [32]. We speculated that its suppression likely represents a feedback response to intracellular lipid accumulation, probably driven by N/MP uptake or impaired lipid clearance. Conversely, PPARG induction could indicate an adaptive effort to mitigate lipotoxicity via enhanced fatty acid uptake, storage, and anti-inflammatory signaling [33]. This metabolic reprogramming marks a shift from anabolic lipid synthesis to stress-responsive lipid handling. However, PPARG-mediated lipid storage cannot substitute for the structural roles of cholesterol in epithelial membranes. Reduced SREBF2 activity may compromise membrane architecture, particularly in keratinocytes where cholesterol is crucial for bilayer stability, fluidity, and lipid raft integrity [34]. Indeed, these cholesterol-rich microdomains support cell adhesion, immune receptor localization, and barrier function. Thus, we hypothesized that SREBF2 downregulation may render vaginal keratinocytes more vulnerable to membrane fragility, impaired signaling, and increased permeability, which is particularly critical in the vaginal mucosa, where epithelial integrity underpins host defense and immune homeostasis. This, indeed, could disrupt the function of membrane-associated proteins, such as toll-like and growth factor receptors, exacerbating the inflammatory and regenerative impairments induced by N/MPs.

Interestingly, the transcriptional dysregulation of genes implicated in lipid metabolism included, in addition to PPARG and SREBF2, several other key regulators of LD biogenesis (SLC27A1, ACSF3, APOC3, APOA2, and ACADL), suggesting activation of LDs as a protective mechanism against various stresses, including oxidative and ER stress. Indeed, under stress conditions, increased free fatty acid (FFA) levels trigger LD formation to prevent lipotoxicity and maintain membrane homeostasis. LDs also sequester vulnerable lipids like PUFAs, protecting them from damage. However, prolonged accumulation of FFA can worsen cellular stress, creating a cycle that further promotes LD biogenesis [35]. Based on the modulation of these genes, we assumed that the perinuclear vesicles accumulating in vaginal keratinocytes following PE N/MP exposure could be not solely endosomes, but potentially LDs enriched in neutral lipids. Oil Red O staining confirmed this hypothesis in VK2 E6/E7 exposed to PE QDs/NPs and PE N/MPs, revealing that the LDs primarily consisted of neutral lipids, such as triglycerides and cholesteryl esters [36]. Neutral LDs have occasionally been observed in keratinocytes under both normal and pathological conditions [37, 38]. Under physiological conditions, keratinocytes synthesize and export lipids to maintain the skin’s permeability barrier. Whenever a skin irritant compromises the membrane barrier and increases its permeability, LDs accumulate as a protective response to support the restoration of cellular homeostasis and so facilitate membrane repair [37].

Immune-related transcriptional changes revealed a parallel axis of vulnerability in vaginal keratinocytes exposed to N/MPs. Increased expression of CD4, CD8A/B, ZAP70, LCK, and ITK, alongside immunoregulatory genes (IDO1, CTLA4, TIGIT, FOXP3), might indicate a dual immune response involving both cytotoxic activation and immune suppression. In line with this, IL-6 secretion was elevated at low PE N/MP doses, whereas IL-10 protein levels were higher at the highest dose of PE N/MPs, despite transcript induction at intermediate concentrations. Together, these findings indicate that low-dose PE N/MP exposure may prime adaptive signaling responses, while higher doses elicit functional stress and activate downstream immunoregulatory mechanisms, highlighting a dose-dependent modulation of epithelial immune activity.

Moreover, direct activation of keratinocytes, capable of producing a full range of pro-inflammatory cytokines, can strongly influence local and recruited immune cells, triggering rapid and pronounced skin responses to noxious agents [39]. We hypothesized that, in the vaginal mucosa, such dysregulation may manifest as local lymphocyte and macrophage activation coupled with regulatory T cell-mediated tolerance, this potentially hindering pathogen clearance and immune surveillance, promoting persistent infections, such as human papillomavirus (HPV), and increasing the risk of cervical intraepithelial neoplasia or carcinoma. These findings align with growing evidence of immune and microecological dysregulation in HPV pathogenesis [40].

Furthermore, enhanced ROS production via CYP and NOX enzymes may cause DNA damage and mitochondrial stress, fostering mutagenesis and architectural disruption through epithelial fibrosis and extracellular matrix (ECM) remodeling, involving genes, such as COL6A1, LAMA4, and PDGFB [41]. Functional assays revealed that, despite widespread transcriptional modulation of redox-related genes, a significant accumulation of H₂O₂ was detected only at the highest PE N/MP concentration. This finding may indicate that low-dose exposure elicits transcriptional redox responses that remain effectively buffered and do not translate into overt oxidative stress, whereas high-dose exposure exceeds cellular antioxidant capacity, resulting in measurable ROS accumulation.

GSEA further confirmed a consistent transcriptional signature in vaginal keratinocytes exposed to both low and high N/MP concentrations. Despite dose differences, several biological processes were commonly enriched, indicating core cellular programs activated in response to toxic stress. The enrichment of “proteinogenic amino acid metabolism” and Reactome’s “metabolism of amino acids and derivatives” terms suggest a shift toward catabolic energy production, enabling keratinocytes to meet altered energy and redox demands under stress [42]. Concurrently, enrichment of oxidative stress pathways, including “responses to oxygen-containing compounds” and “oxidoreductase activity”, mirrors a persistent pro-oxidant state, potentially affecting DNA integrity, protein structure, and lipid stability. Additionally, GSEA revealed enrichment of pathways governing cell adhesion and epithelial cohesion. Terms, such as “cell-cell adhesion”, “regulation of cell adhesion,” and “cell adhesion molecule binding” point to N/MP-induced disruption of epithelial architecture, likely linked to altered expression of structural genes, such as SREBF2 [34]. Finally, the enrichment of immune-related pathways, including “lymphocyte activation”, “regulation of immune response”, and “inflammatory response”, underscores the simultaneous activation of pro-inflammatory and regulatory pathways that may impair mucosal defense, promote pathogen persistence, and disrupt epithelial homeostasis, with lipid composition playing a key role in maintaining immune and microbial balance [43].

By the combined use of synchrotron radiation-based STXM and LEXRF, we also were able to demonstrate the internalization and intracellular localization of the PE QDs/NPs, as revealed by the discrete cytoplasmic presence of Se hotspots. Specifically, STXM Abs and PhC imaging revealed marked morphological differences between control and NP-exposed cells. Control cells retained typical nuclear morphology with higher absorption in the nucleus, while PE QD/NP-treated cells showed bright vesicular structures, consistent with vesicle formation and potentially endosomal/lysosomal accumulation of particles. On the other hand, LEXRF analysis indicated significant perturbations in the elemental composition, mainly C, O, Na, and Mg, in VK2 E6/E7 cells exposed to PE QDs/NPs, this suggesting the impact of PE in the osmotic equilibrium and cell volume control as well as activation of oxidative stress and inflammatory pathways. Indeed, even though this requires further investigation, Na imbalance can disrupt cellular osmolarity and volume control, while Mg, an essential cofactor for ATP-dependent enzymatic processes, is critical for maintaining genomic stability and cellular homeostasis [44]. Mg depletion has been associated with impaired DNA repair mechanisms and the activation of inflammatory pathways [45]. As part of a stress or inflammatory response, keratinocytes may upregulate the synthesis of mucins and other glycoproteins [46], which are C-rich components of the extracellular environment. Noteworthy, while C enrichment could be related to the PE N/MPs, its redistribution, which is particularly pronounced with the PE 250 dose, may also support intracellular LD accumulation, reinforcing the NanoString transcriptional evidence that point to metabolic reprogramming, including lipid metabolism dysregulation. The technologies we employed cannot discriminate between exogenous polymer-derived C and endogenous C-rich biomolecules, so further analytical methods would be required to resolve C accumulation. Altogether, our multi-model evidence strengthens the biological relevance of PE N/MP exposure in human vaginal keratinocytes, supporting both structural and metabolic alterations, which align with observations reported in other cellular models [12, 30]. Importantly, synchrotron-based elemental mapping and transcriptomic profiling provide complementary but non-causal layers of information. While elemental redistribution reflects structural and physicochemical perturbations, transcriptional changes capture downstream adaptive responses. Their convergence supports a coherent stress-response framework rather than a direct one-to-one causal relationship.

Based on pathway-level analyses, it is plausible that sustained metabolic stress could influence epithelial homeostasis. However, potential downstream consequences, such as epithelial barrier weakening, altered immune surveillance or increased susceptibility to infection, remain hypothetical and were not directly assessed in the present study. Accordingly, these aspects should be regarded as predicted biological outcomes that warrant targeted functional investigation in polarized epithelial cultures, co-culture systems, or in vivo models.

Importantly, the in vitro exposure conditions used here reflect acute cellular stress paradigms and cannot be directly extrapolated to real-world vaginal exposure scenarios. Although recent work has demonstrated that disposable intravaginal period products can release billions of NPs under conditions that mimic vaginal fluid exposure [15], indicating that human exposure to PE and other polymer N/MPs is plausible during product use, the concentration, duration, and cumulative burden of PE N/MP exposure required to elicit comparable effects in vivo remain unknown. Moreover, while our study focused on well-defined PE micro- and nanospheres to ensure experimental reproducibility and mechanistic clarity, additional research is warranted to explore the effects of alternative physical forms of N/MPs, such as fibers or irregular fragments. These more complex and heterogeneous shapes (commonly released from personal care products and present in real-world environmental matrices) could differently interact with epithelial cells and potentially elicit distinct or complementary biological responses. Comparisons with other epithelial models suggest that particle-induced responses could be context-dependent and tissue-specific. Indeed, our previous work showed that acute exposure to PE N/MPs did not significantly affect viability or proliferation in bronchial and alveolar epithelial cells (BEAS-2B and A549), aside from subtle cytoskeletal alterations [9], whereas in VK2 E6/E7 vaginal epithelial cells, significant effects on cell viability and morphology were observed [16]. As regards polymer-specific responses, a systematic evaluation of other types of polymers will require standardized, biologically validated particle preparations and is beyond the scope of the present study.

A limitation of this study is the use of VK2 E6/E7 immortalized vaginal keratinocytes expressing HPV E6 and E7 oncogenes, which may interfere with p53 and Rb signaling. This might shape the magnitude or threshold of stress-adaptive responses elicited upon PE N/MP exposure. Importantly, however, the conclusions of the present study are derived from comparative analyses within the same cellular background, rather than from absolute comparisons to primary or HPV-negative epithelial cells. All experimental conditions were therefore equally influenced by E6/E7 expression, preserving the internal validity of PE size- and dose-dependent effects. Noteworthy, the observation that PE N/MP exposure exacerbates metabolic and oxidative stress signatures in a cellular context already characterized by altered p53/Rb signaling may be biologically relevant rather than artefactual. Such amplification of stress-adaptive or maladaptive pathways could potentially aggravate pre-existing epithelial vulnerability, a scenario that may be particularly pertinent in chronically stressed or transformed tissues. Nevertheless, we acknowledge that E6/E7-mediated pathway modulation may limit direct extrapolation to primary vaginal epithelium, and future studies employing primary keratinocytes or organotypic models will be important to further validate tissue-level relevance.

Moreover, physiological and hormonal variables known to modulate vaginal epithelial integrity—such as menstrual cycle phase, progestin use, menopause, and microbiota composition—were not addressed and may significantly influence susceptibility to environmental stressors.

Collectively, our findings should be interpreted as mechanistic and hypothesis-generating, providing a cellular framework to guide future studies investigating the long-term relevance of plastic particle exposure for female genital epithelial biology. Establishing direct links to disease outcomes will require integration of functional barrier assays, immune readouts, and epidemiological or biomonitoring data.

Finally, by highlighting the risks associated with potential N/MP release from certain feminine hygiene products, our study underscores an urgent need for safer, biodegradable alternatives, representing a simple yet powerful preventive measure to safeguard women’s reproductive health by reducing N/MP exposure at the source.

## Materials and Methods

### Nano/microplastics and reagents

The PE nano- and microspheres mixture, with sizes ranging from 200 to 9900 nm, was commercially sourced from Cospheric LLC (Santa Barbara, CA, USA). According to the manufacturer’s specifications, the material consists of > 90% spherical PE particles, with a reported particle number of approximately 2.44 × 10¹¹ particles per gram (assuming a mean diameter of 2 μm) and a density of 0.95–0.98 g/cm³. Size distribution and morphology were verified by the supplier using scanning electron microscopy (SEM), and representative SEM images were provided by Cospheric LCC. These manufacturer-certified specifications were used to ensure batch consistency and reproducibility. The product was produced and packaged in a controlled environment. Prior to use in cell culture experiments, PE particles were tested for sterility to exclude microbial contamination. No evidence of contamination was detected under the experimental conditions employed. Particles were subsequently handled and dispersed under sterile laboratory conditions. PE N/MPs were suspended in a 1% Tween20 solution (Dako, Agilent Technologies, Santa Clara, CA, USA) to create a 100 mg/mL stock, following the supplier’s instructions. Working dilutions were prepared using ultrapure distilled water to ensure that the final Tween20 concentration in the cell culture medium remained below 0.01%, thereby limiting potential cytotoxic effects associated with the surfactant [47]. All stock and working solutions were thoroughly vortexed before use. In cell culture assays, PE N/MPs were used at concentrations of 25, 50, and 250 μg/mL. To rule out any confounding effects of the Veh, a control containing the same amount of Tween20 as in the highest N/MPs concentration was included in all experiments. Additionally, fluorescently labeled PE nanoparticles (PE QDs/NPs, 200–300 nm size range) were provided by the Joint Research Center (JRC) in Ispra, Italy [21]. PE QDs/NPs were prepared using an oil-in-water emulsion method. Specifically, 30 mg of PE polymer (Sigma-Aldrich, St. Louis, MO, USA) was dissolved in 3 g of toluene by heating to 100 °C for one hour. A volume of 200 µL of Qdot™ 655 ITK™ CdSe QDs (Thermo Fisher Scientific, Waltham, MA, USA) was then added to the organic phase. Separately, 27 mL of boiling MilliQ water containing 7.5 mg of sodium cholate (Sigma-Aldrich, St. Louis, MO, USA) was prepared as the aqueous phase. The two phases were combined and emulsified using an Ultra-Turrax T25 homogenizer (IKA-Werke GmbH & Co., Staufen, Germany) at 16,000 rpm for 2 min, then ultrasonicated with a Vibra-Cell ultrasonic processor (Sonics & Materials Inc., Newtown, CT, USA) at 40% amplitude for 2.5 min, followed by rapid cooling in an ice-water bath for 3 min. The resulting suspension was filtered through 5 µm polyether-sulfone membrane syringe filters to achieve the desired nanoparticle size. Residual organic solvent was removed using a rotary evaporator, and the final yield was calculated by weighing the freeze-dried material. TEM imaging confirmed heterogeneous PE particles with a broad size distribution and frequent clustering. DLS analysis yielded a Z-average hydrodynamic diameter of ~230 nm and a low Polydispersity Index (PDI ~ 0.12), measured using a Zetasizer Lab Blue (Malvern Panalytical, Malvern, UK) (Additional File 1: Fig. 3). The labeled nanoparticles were handled under sterile conditions, dispersed in sterile Milli-Q water and stored in the dark at 4 °C. Furthermore, they were tested to exclude microbial contamination in cell culture medium before experimental use.

### Cell Cultures and Treatments

The VK2 E6/E7 cell line (ATCC CRL-2616), established from normal vaginal epithelial tissue of a premenopausal woman undergoing anterior–posterior vaginal repair surgery, was selected as it represents the only commercially available model of healthy vaginal epithelium. Cells were cultured in Keratinocyte Serum-Free Medium (K-SFM; GIBCO, Carlsbad, CA, USA), supplemented with 0.1 ng/mL human recombinant epidermal growth factor (EGF), 0.05 mg/mL bovine pituitary extract, 44.1 mg/L calcium chloride (final concentration 0.4 mM), and 1% penicillin/streptomycin (Aurogene, Rome, Italy). Cultures were maintained at 37 °C in a humidified atmosphere containing 5% CO₂. Sub-culturing was routinely performed at 80% confluence to prevent spontaneous differentiation, which keratinocytes undergo at higher densities. All cell cultures were routinely screened and confirmed to be free of mycoplasma contamination before starting experiments. Microscopic images of treated cells were acquired at 40× magnification using the EVOS XL Core Imaging System (Thermo Fisher Scientific, Waltham, MA, USA). As for gene expression experiments, 1,5 × 10^5^ cells/ well were seeded onto a 6-well plate and treated with Veh (0.0025% Tween20) or PE N/MPs at final concentrations of 25 and 250 μg/mL in 2 mL of complete growth medium for 48 h. For synchrotron acquisitions, VK2 E6/E7 cells were seeded onto Si₃N₄ membranes with a thickness of 200 nm (Silson Ltd, Warwickshire, UK), placed in 24-well culture plates (2 × 10⁴ cells/ well). The cells were then incubated with 500 μL complete growth medium containing either unlabeled PE N/MPs (at final concentrations of 25, 50, and 250 μg/mL) or PE QDs NPs (at final concentrations of 25 and 50 μg/mL) for 48 h. Control samples were cultured under identical conditions but were not exposed to nanoparticles.

### Gene Expression Analysis via NanoString Technology

Total RNA was isolated from VK2 E6/E7 cells and purified using a combination of the Total RNA Purification Kit, the RNase-Free DNase I Kit, and the RNA Clean-Up and Concentration Kit (all from Norgen Biotek Corp, Thorold, ON, Canada). The RNA quantity and purity were assessed using a NanoDrop 2000c spectrophotometer (Thermo Fisher Scientific, Waltham, MA, USA), and samples were stored at −80 °C until further analysis. Gene expression profiling was performed on three independent biological replicates using the NanoString nCounter System with the nCounter Human Metabolic Pathways Panel (NanoString Technologies, Seattle, WA, USA), which includes probes for 768 genes associated with over 30 metabolic pathways, as well as 20 internal reference genes for normalization, as previously described [48]. For each sample, 100 ng of total RNA was hybridized with capture and reporter probe sets in hybridization buffer at 65 °C for 18 h, following the nCounter XT CodeSet Gene Expression Assay protocol. Post-hybridization, the samples were processed using the nCounter Prep Station, and data acquisition was carried out via the nCounter Digital Analyzer, according to the manufacturer’s instructions. Raw data were analyzed using the nSolver Analysis Software 4.0, which performed quality control and normalization based on the geometric mean of negative controls, internal positive controls, and housekeeping genes showing a coefficient of variation (% CV) below 25% across all replicates. Differential gene expression was determined by applying a log_2_ fold change cut-off of > 1 or < -1, comparing cells exposed to 25 or 250 µg/mL PE N/MPs with those treated with Veh. Statistical significance has been assessed by one-way ANOVA corrected for multiple comparisons using Tukey’s test. To visualize key transcriptional changes in metabolic pathways, a clustered heatmap was generated using the ggplot2 package in R (version 4.2.3), illustrating expression profiles relative to Veh-treated controls under different exposure conditions. GSEA has been performed using GSEA (https://www.gsea-msigdb.org/gsea/index.jsp, accessed on 26/06/2025) [49] using Hallmark (h), Curated gene sets (C2), and Ontology gene sets (C5). GSEA calculates significant enrichment by false discovery rate (FDR) based on the Benjamini-Hochberg test. Only classes with a FDR q-value < 0.05 have been considered significantly enriched. Bubble plots of enriched terms from GSEA were generated in R using custom scripts.

### ROS detection

VK2 E6/E7 cells were seeded in a 96-well plate (6 ×10^3^ cells/well in 80 μL of complete growth medium) and exposed to PE N/MPs or Veh for 48 h. ROS-Glo™ H₂O₂ Assay (Promega, Madison, WI, USA) was then performed directly on the supernatants, following the manufacturer’s non-lytic protocol. The H₂O₂ substrate solution was added to each well to a final volume of 100 μL, and the plate was incubated at 37 °C in a 5% CO₂ incubator for 3 h. After incubation, the supernatants were transferred to an opaque white 96-well plate and 100 μL of the ROS-Glo™ detection solution was added to each well. Following an additional 20-min incubation at room temperature, luminescence was measured using a GloMax® Discover luminometer (Promega, Madison, WI, USA). Control wells containing medium alone with either Veh, PE 25 or PE 250 were included to normalize luminescence readings. The experiment was performed in three independent biological replicates, each with six technical replicates per point.

### ELISA for cytokine quantification

VK2 E6/E7 cells were seeded at a density of 1.5 × 10⁵ cells per well in 6-well plates and treated with Veh or PE N/MPs in 2 mL of complete growth medium for 48 h. After treatment, conditioned media were collected and centrifuged at 300 × g to remove cellular debris. The concentrations of IL-6 and IL-10 in the supernatants were quantified using human Quantikine^TM^ ELISA kits (R&D Systems, Minneapolis, MN, USA) as previously described [50]. Recombinant cytokine standards supplied with each kit were serially diluted to generate standard curves. Standards and samples (200 µL each) were added to pre-coated 96-well plates and incubated at room temperature. Plates were then washed and incubated with horseradish peroxidase-conjugated detection antibodies, followed by additional washing steps and the addition of substrate solution. The reaction was stopped with stop solution, and absorbance was measured at 450 nm using a microplate reader (Bio-Rad, Hercules, CA, USA). Cytokine concentrations were calculated from the standard curves and expressed as pg/mL. The experiment was performed in three biological replicates, with three technical replicates per point.

### Fluorescence microscopy

The Si₃N₄ windows were placed on the sample holder of a Zeiss LSM 900 confocal laser scanning microscope (Carl Zeiss GmbH, Jena, Germany), which operated in fluorescence mode to detect the red (Texas Red filter: excitation at 560 ± 55 nm and emission at 645 ± 75 nm) and green (Alexa Fluor 488: excitation at 495 ± 20 nm, emission at 519 ± 25 nm) channels. Images were captured at 60× magnifications for each condition, and image processing was performed using FiJi software [51]. For each experimental setup, at least three to four cells per condition were preselected using fluorescence light microscopy prior to synchrotron-based X-ray microscopy analysis. Cells were selected based on objective and predefined criteria, including intact morphology, adherence to the substrate, absence of overt cell death or fragmentation, and suitable thickness for soft X-ray transmission.

### Synchrotron radiation-based STXM and LEXRF

Following incubation with unlabeled PE N/MPs or PE QDs/NPs, cells were fixed at room temperature using a 4% aqueous paraformaldehyde solution (Thermo Fisher Scientific, Waltham, MA, USA) for 20 min in the dark, then washed with phosphate-buffered saline (PBS) and Milli-Q water, and the membranes were left to dry overnight. Subsequently, the samples were analyzed by STXM and LEXRF at the TwinMic beamline of the Elettra Sincrotrone Trieste facility (Trieste, Italy) [52]. The TwinMic microscope was operated in scanning transmission mode, where a multilayer Au zone plate diffractive optics, with a 600 μm diameter and 50 nm outermost zone width, focused the monochromatic X-ray beam onto the sample, producing a probe size ranging from micrometric to sub-micrometric scale. Samples were raster-scanned perpendicularly to the incoming beam while a fast-readout CCD camera collected the transmitted X-rays [53], and an eight-silicon drift detector-based XRF system simultaneously detected emitted fluorescence photons [54]. Abs and PhC images revealed morphological details at sub-micrometric resolution, while LEXRF provided elemental distribution maps correlated with the morphology. For XRF mapping, parameters were adjusted to a photon energy of 1.7 keV to optimize Se excitation, with a spot size of  500 nm, a dwell time of 5 seconds per pixel for XRF acquisition, and a CCD dwell time of 60 ms for STXM imaging. Each elemental map required approximately 4–6 h, depending on the scanned area size, with typically two to three cells mapped per Si₃N₄ membrane window. Elemental maps were generated and processed using PyMCA software [55] by deconvolving and fitting the XRF spectra and were plotted with the XRFitVis web application [56]. Spectral fitting was performed with the Hypermet model, including background subtraction by the Sensitive Nonlinear Iterative Peak (SNIP) algorithm (SNIP width = 135). Emission lines were fitted as Gaussian peaks with automatically optimized widths. Elemental distributions were analyzed using identical acquisition settings across samples; beam intensity was monitored throughout acquisition and used for normalization when necessary. Accordingly, elemental maps were employed for qualitative and semi-quantitative assessment of relative signal intensity and intracellular spatial redistribution rather than absolute elemental quantification. Although Abs and differential PhC images can be acquired simultaneously with XRF maps, in order to increase the absorption contrast and better identify morphological features, high-resolution STXM imaging was performed at a lower photon energy, namely 1.5 keV, with a spot size of approximately 250 nm and a dwell time of 25 ms per pixel. Synchrotron radiation-based analyses were performed using three independent biological replicates, which were acquired within the same experimental session.

### Oil Red O staining

VK2 E6/E7 cells exposed to PE N/MPs and PE QDs/NPs were fixed in 10% formalin for 45 min at room temperature, then briefly incubated in 60% isopropanol for 5 min. Subsequently, they were stained with Oil Red O solution (Sigma-Aldrich, St. Louis, MO, USA) for 5 min. Images were captured at 40x and 20x magnification using the EVOS XL Core Imaging System (Thermo Fisher Scientific, Waltham, MA, USA). To quantify lipid content, the stained LDs were eluted with isopropanol, and absorbance was measured at 490 nm with an ELISA Microplate Reader (Bio-Rad, Hercules, CA, USA), as previously described [57]. Image acquisition was performed using identical microscope settings across all conditions, background signal was subtracted uniformly, and quantification was carried out by elution-based absorbance measurement, a widely used and reproducible approach for LD assessment. While the analysis was not performed in a blinded manner, objective spectrophotometric quantification was used to minimize operator-dependent bias. For quantification, Oil Red O staining in PE N/MPs cells was normalized to Veh-treated controls, while for PE QDs/NPs it was normalized to unexposed controls, which were set as reference (value = 100%). The experiment was performed in three independent biological replicates, each analyzed in two technical replicates.

### Statistics

Statistical analyses were conducted using GraphPad Prism version 8.0.1 (GraphPad Software Inc., San Diego, CA, USA). Data are presented as mean ± standard deviation (SD) from at least three independent biological replicates. Parametric tests were applied based on the assumption of approximate normal distribution, as commonly accepted for cell culture studies. Comparisons between two experimental groups were performed using two-tailed unpaired Student’s t-tests. Comparisons involving more than two groups were conducted using one-way ANOVA followed by Tukey’s post hoc test to correct for multiple comparisons. Variances between groups were assumed to be similar based on experimental design and inspection of the data. No data points were excluded from the analyses. Differences were considered statistically significant when *p*-values were < 0.05.

## Supplementary information

Supplementary Information

## Data Availability

The datasets used and/or analyzed during the current study are available from the corresponding author on reasonable request.
