# Supplementary material for: Polyethylene nano- and microplastics trigger metabolic stress responses in human vaginal epithelial cells
Source: Cell Death Discov. 2026 Mar 24;12:173. doi: 10.1038/s41420-026-03038-6 (PMC13039758; doi:10.1038/s41420-026-03038-6)
Supplement: Supplementary file 1 — Supplementary Information [file 41420_2026_3038_MOESM1_ESM.pdf]

# Figure S1

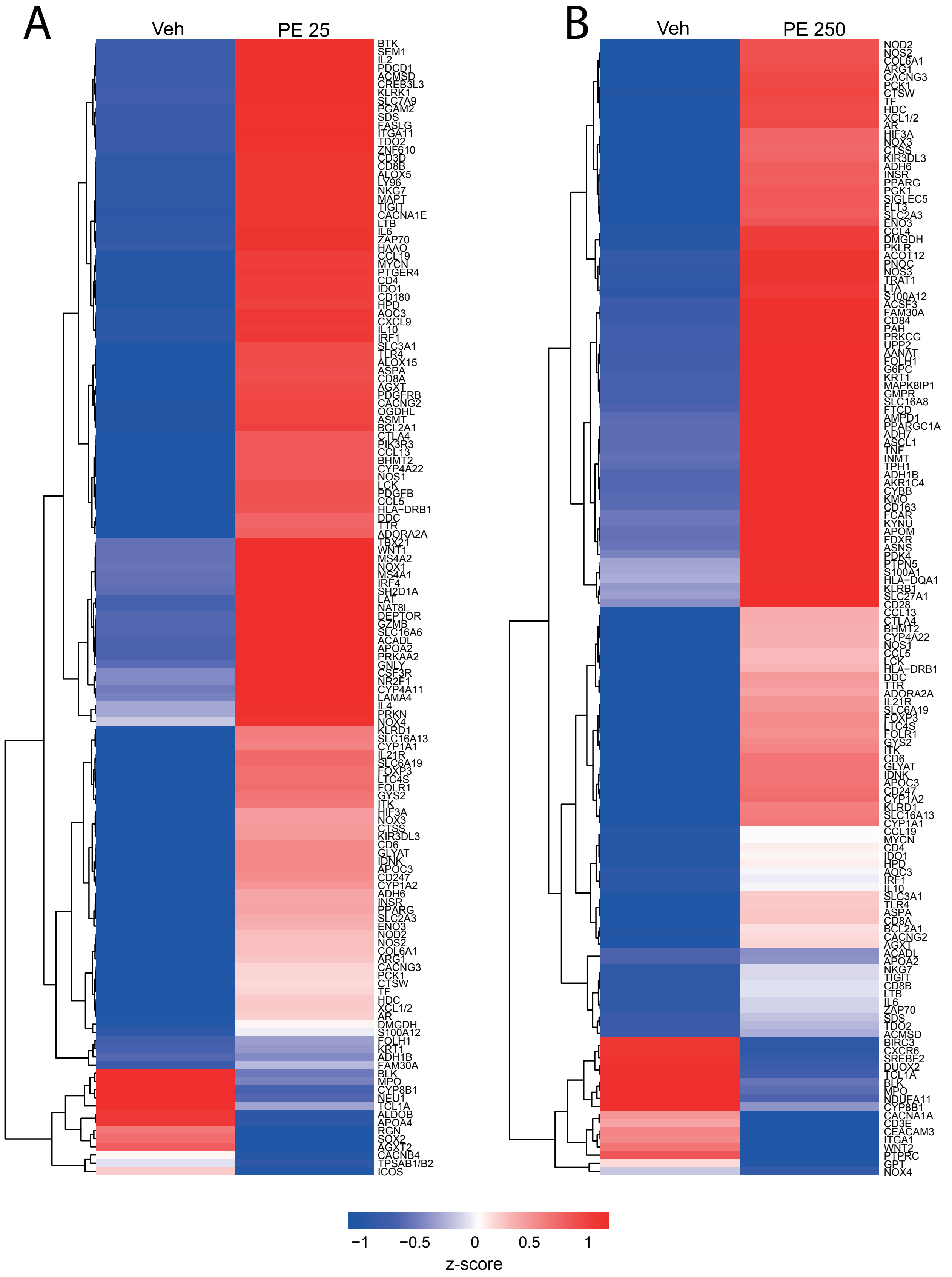


**Figure S1.** PE N/MPs exposure primarily promotes gene transcription in human immortalized vaginal keratinocytes. **A.** Cluster heatmap of modulated genes in VK2 E6/E7 human immortalized vaginal keratinocytes treated with 25 µg/mL (PE 25) of a mixture of PE nano- and microspheres (200 nm - 9 µm) for 48 hours. **B.** Cluster heatmap of modulated genes in VK2 E6/E7 human immortalized vaginal keratinocytes treated with 250 µg/mL (PE 250) of PE nano- and microspheres for 48 hours. Data are expressed as z-score, the color legend is provided in the figure.

# Figure S2

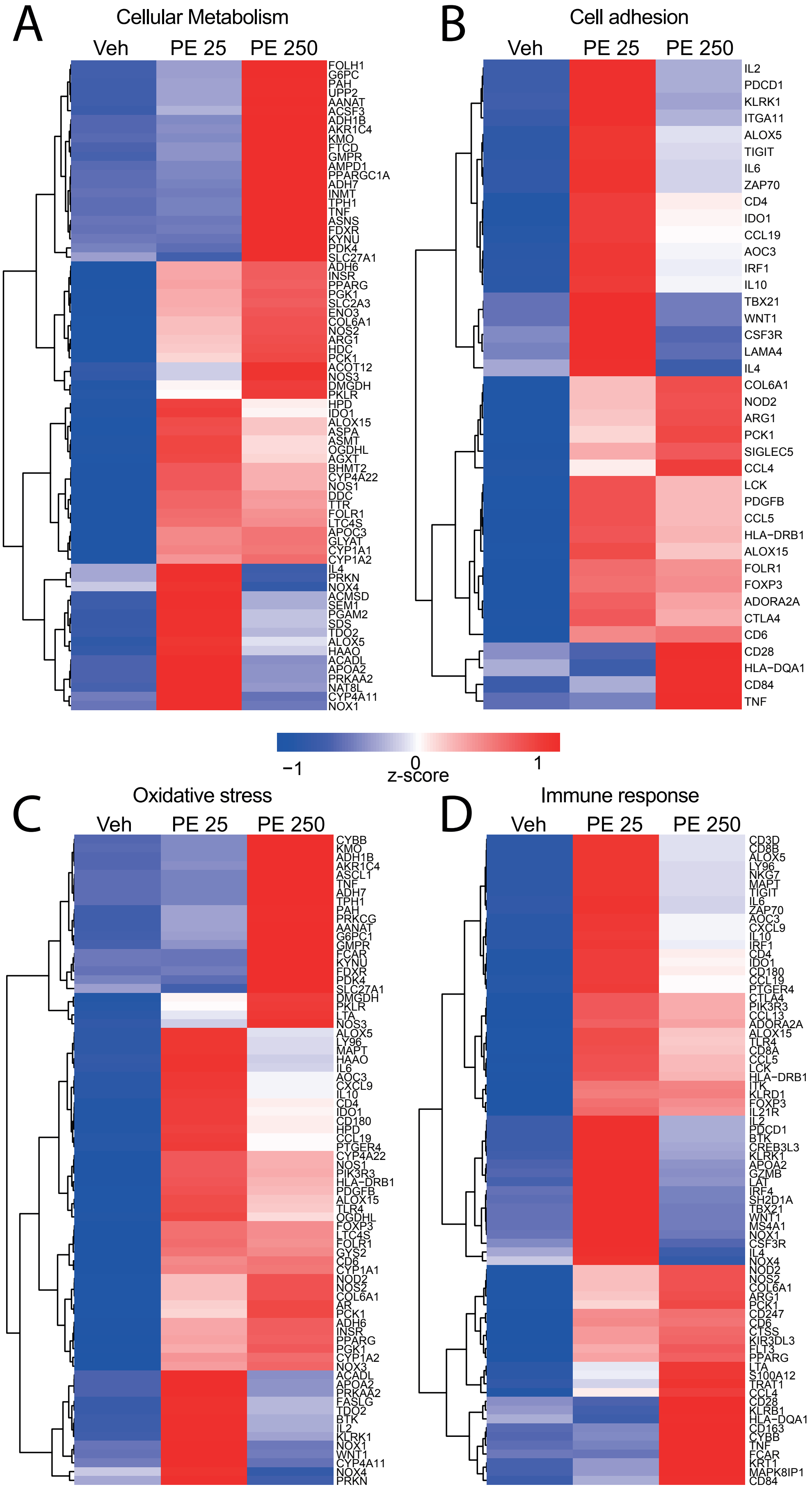


**Figure S2.** PE N/MPs upregulates genes involved in cellular metabolism, adhesion, oxidative stress and immune response. **A.** Cluster heatmap of upregulated genes involved in cellular metabolism in VK2 E6/E7 cells treated with 25 μg/mL (PE 25) or 250 μg/mL (PE 250) for 48 hours. **B.** Cluster

heatmap of upregulated genes involved in cell adhesion in VK2 E6/E7 treated as in A. **C.** Cluster heatmap of upregulated genes involved in oxidative stress in VK2 E6/E7 cells treated as in A. **D.** Cluster heatmap of upregulated genes involved in immune response in VK2 E6/E7 treated as in A. Data are expressed as z-score, the color legend is provided in the figure.

# Figure S3

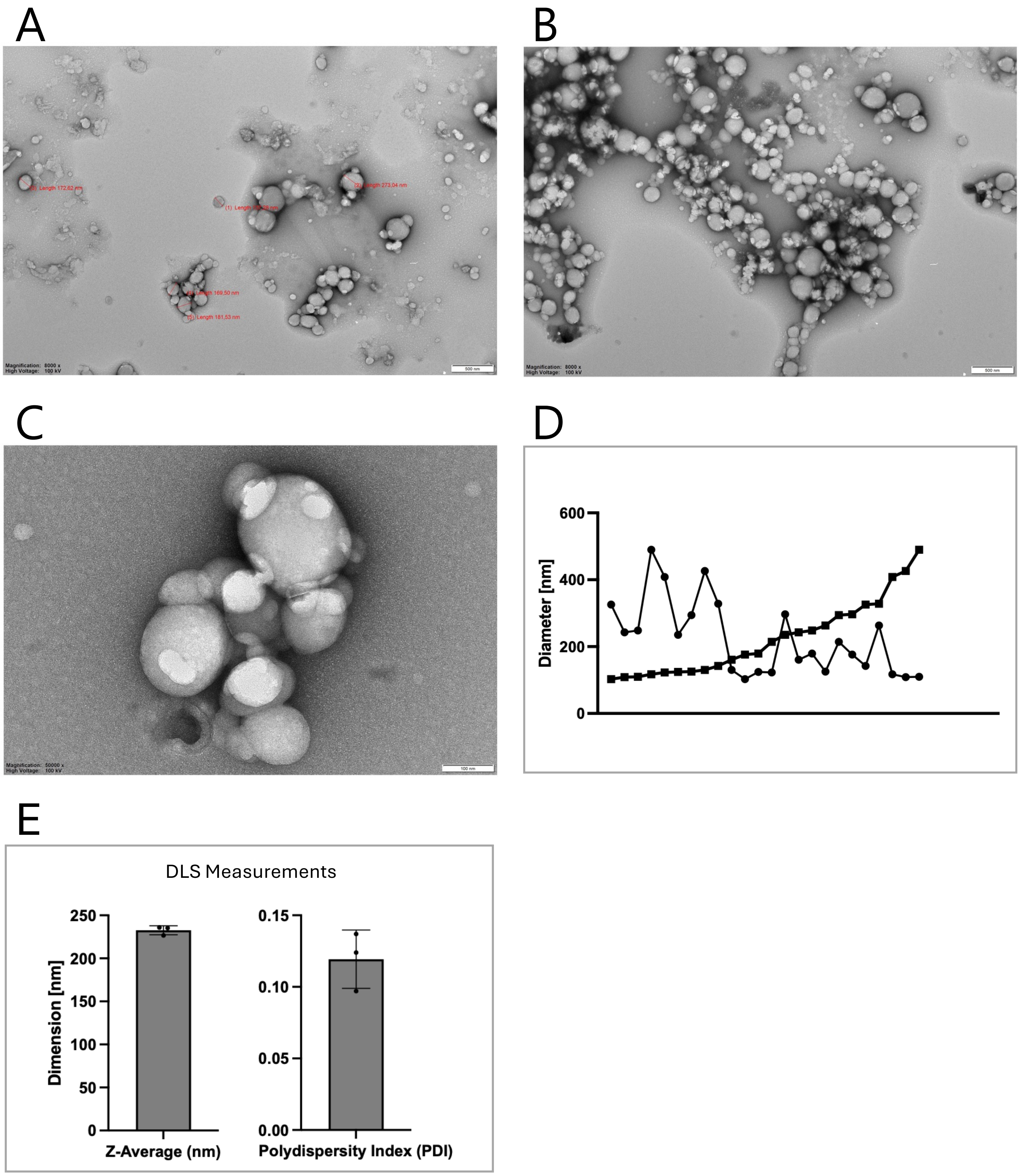


**Figure S3.** Morphological and size characterization of PE QD/NPs. **A-B.** Representative transmission electron microscopy (TEM) images of PE QD/NPs, showing heterogeneous particle morphology and size distribution. Scale bar: 500 nm. In panel A representative particle diameters are highlighted in red. **C.** High-magnification TEM image illustrating nanoscale features and particle aggregation. Scale bar: 100 nm. **D.** Quantitative analysis of PE QD/NPs size distribution based on TEM measurements, with individual particle diameters plotted and corresponding size trend. **E.** Dynamic light scattering (DLS) characterization of representative PE OD/NPs samples reporting L-average hydrodynamic diameter and polydispersity 1ndex (PDI).
